# Functional Activity of Four Autochthonous Strains *L. paraplantarum* AB362736.1, *L. plantarum* MF369875.1, *W. paramesenteroides* CP023501.1, and *E. faecalis* HQ802261.1 in a Probiotic Grape Marmalade during Storage

**DOI:** 10.3390/antiox8060165

**Published:** 2019-06-06

**Authors:** Lesly Samedi, Albert Linton Charles

**Affiliations:** Department of Tropical Agriculture and International Cooperation, National Pingtung University of Science and Technology, 1 Shuefu Road, Neipu, Pingtung 912 01, Taiwan; leslysamedi3@gmail.com

**Keywords:** antioxidative properties, 2,2-diphenyl-1-picrylhydrazyl (DPPH), grape marmalade, *Lactobacillus*, probiotics

## Abstract

Grape foods with probiotics are sources of beneficial bacteria for the gastrointestinal (GI) tract and also have a high antioxidant capacity. The addition of probiotics to dairy food is a traditional process; therefore, probiotic non-dairy products might contribute to a daily antioxidant diet to improve consumer life quality and health. This research was undertaken to develop a grape marmalade with a probiotic base to investigate the potential antioxidant activity in the probiotic non-dairy product. Thus, changes in active culture numbers, pH level, glucose concentration, and antioxidant properties were evaluated. Most of the isolates demonstrated higher growth in the grape marmalade than the synthetic grape marmalade, which was greater than 7 log colony-forming units (CFU)/g within 90 days of storage at 4 °C. In addition, most of the wild isolates grew beyond the critical count of 10^6^ CFU/g in sampling between 60 and 90 days of storage. Moreover, probiotic grape marmalade with probiotics showed a strong antioxidant capacity that failed to differ significantly with the synthetic medium. The study confirmed *Lactobacillus paraplantarum* AB362736.1, *Lactobacillus plantarum* MF369875.1, *Weissella paramesenteroides* CP023501.1, and *Enterococcus faecalis* HQ802261.1 were ideal bacteria for the probiotic process of grape marmalade.

## 1. Introduction

Probiotics are live microorganisms, which, when administered in adequate amounts, confer a health benefit on the host [1]. They can also be carefully selected and added to the diet according to their health benefits. However, not all microorganisms are probiotics. To earn this label, they must prove their health benefits in a sufficient concentration allowing them to survive the acidity of the stomach to be able to act in the intestines. Most probiotics currently used are *Lactobacillus* and *Bifidobacterium* [2].

Today, there is a growing demand for both non-dairy and dairy probiotic products in the market, as well as bacteria added to beverages marketed as supplements, tablets, and freeze-dried preparations [3]. Probiotics added to a new food or beverage lead to many substantial variables to be examined to ensure viability, which is important for safety. Moreover, they must survive and preserve their functionality in storage, and also in the foods into which they are mixed without producing off-flavors. It should be noted that the physiological condition of the bacteria incorporated into products is of great significance and, therefore, relies greatly on three basic parameters: the time of harvest of the bacteria, whether in the midst of the log phase or stationary growth phrase; the conditions resulting in static period on the handling of bacteria during and after harvesting; and the structure of the growth environment with regard to product constitution into which they may be incorporated [4]. Furthermore, fermented dairy and non-dairy products could include a shelf life such that they have a high viable bacteria count of at consumption, averaging a lower limit of 10^6^ colony forming units (CFU)/g of product [5].

Long ago, probiotics were incorporated into yogurts and dairy products; however, the cholesterol content and lactose intolerance remained two major drawbacks related to their consumption for some people. Furthermore, fruits and vegetables do not have dairy allergens that may prevent consumption by certain segments of the population [3]. Grapes are a non-climacteric type of fruit, generally occurring in clusters which are rich in vitamins, minerals, dietary fibers, and polyphenols in particular [6]. Hence, the objectives of this research were to develop a grape marmalade with a probiotic base, to investigate the functional activity of the probiotic grape marmalade, and to evaluate the viability of the applied bacteria strains in the marmalade during 90 days of storage at both room and refrigerated temperatures. The selected *Lactobacillus* isolates *L. paraplantarum* AB362736.1, *Lactobacillus plantarum* MF369875.1, *Weissella paramesenteroides* CP023501.1, and *Enterococcus faecalis* HQ802261.1 were further investigated for their potential antioxidant activity in a non-dairy grape marmalade with a probiotic base.

## 2. Materials and Methods

### 2.1. Grape Control Media (GCM)

For the preparation of the control, a grape medium was prepared to determine the growth and survival of four autochthonous strains of *L. paraplantarum* AB362736.1, *L. plantarum* MF369875.1, *W. paramesenteroides* CP023501.1, and *E. faecalis* HQ802261.1. The medium was formulated as follows: 55 g of sugar, 1.5 g of agar–agar, and 500 mL of condensed grape extract. Food-quality lactic acid was added to adjust the medium to pH 4.5 as described by Randazzo [7]. The marmalade was distributed in sterile containers and maintained at 4 and 25 °C before being inoculated with the bacterial strains.

### 2.2. Grape Marmalade Samples (GM)

The grapes used in this study were provided by a local farm in Pingtung (Taiwan) producing organic grapes (*Vitis vinifera*). The formulation of the jam followed a regular industrial recipe; 83% grapes, 15% sucrose, and 2% lemon juice were mixed then heated to 40–45 °C to obtain a degree Brix (°Bx) value of 40. Then, the marmalade was stored (180-g portions) in glass jars with no additives [8].

### 2.3. Bacterial Strains

The four wild strains used in this study belonged to the microbial collection of the laboratory. The autochthonous isolates were screened for their phenotypic, genotypic, biochemical, and technological attributes. The strains were studied previously for bacterial properties such as resistance to acid and bile, the capability to adapt to the cell line of the intestinal epithelium, antimicrobial activity, lysozyme resistance, activity in HT-29 cells, biofilm synthesis, and antibiotic sensitivity. In addition, the strains were identified as *L. paraplantarum* AB362736.1, *L. plantarum* MF369875.1, *W. paramesenteroides* CP023501.1, and *E. faecalis* HQ802261.1, which are the GenBank numbers for the partial 16S sequence at http://blast.ncbi.nlm.nih.gov [9,10,11]. Additionally, *Lactobacillus rhamnosus* GG ATCC 53103 was used as a control. All isolates were cultured and kept within 20% glycerin at −80 °C for further use.

### 2.4. Preparation and Inoculation of Probiotic Cultures into Grape Control Media and Grape Marmalade

Four different strains isolated from different plant leaves were sub-cultured in MRS medium to 1% (*v*/*v*) inoculum. Bacteria isolates were collected at 10,000 rpm for 10 min, and maintained in a 0.9% NaCl solution, before being diluted 10-fold in the same condensed solution. Then, 100 g of the grape marmalade product was aseptically dispensed into sterilized tubes [12,13]. The products were cultured with fresh probiotic cells, as reported above, to a final density of 10^9^ CFU/g marmalade. The bacterial solution was then mixed into the marmalade and kept at 4 °C and 25 °C. Grape marmalade saturated in salt solution (0.9% *w*/*v* NaCl) was used as control.

### 2.5. Physical and Chemical Tests of the Marmalades

A pH meter was used to evaluate the pH values at regular intervals. The samples were investigated on production day and after 90 days of storage at 4 and 25 °C. To determine the sugar content of marmalade, the d-glucose Enzyme Assay Kit (K-SUFRG) (Megazyme International Ireland Ltd., Wiclkow, Ireland) was used according to the manufacturer’s protocols [14]. Organic acids of Cell-Free Supernatant were determined by HPLC using the ÄKTA Purifier system (GE Healthcare, CA, USA) equipped with an Aminex HPX-87H column (ion exclusion, Biorad, CA, USA) and an ultraviolet (UV) detector operating at 210 nm. Elution was at 60 °C, with a flow rate of 0.6 mL/min, using 10 mM H_2_SO_4_ as the mobile phase. Peaks were identified by comparing migration times and spiking samples with known quantities of standard solutions of acetic acid and lactic acid [9].

#### 2.5.1. Antioxidative Properties of Probiotic Grape Marmalade

The antioxidative capability of the probiotic grape marmalade was evaluated following NO radical, 2,2’-azino-bis(3-ethylbenzothiazoline-6-sulfonic acid) (ABTS), and 2,2-diphenyl-1-picrylhydrazyl (DPPH) radical-scavenging activity assays described below. The probiotic product was centrifuged at 8000× *g* for 5 min at 4 °C, and the supernatants were analyzed after intervals of 0, 24, 48, and 72 h incubation to evaluate their antioxidant properties [15].

#### 2.5.2. Nitric Oxide (NO) Radical

The activity of trapping plant extracts against the NO radical was assessed by the method of Ebrahimzadeh [16]. The generation of nitric oxide was measured from sodium nitroprusside by the Greiss reaction. The amount of nitrite formed was reduced between oxygen and nitric oxide generated by sodium nitroprusside. The percentage of antioxidant activity was estimated from its absorbance at 596 nm (A596 nm) and calculated according to the following formula [17]:

AA% = (100 − ((A596 sample − A596 blank) × 100))/A596 control(1)

where A596 is the absorbance at 596 nm.

#### 2.5.3. ABTS Assay

The ABTS radical-scavenging activity of grape marmalade extracts was evaluated by the ABTS cation decolorization assay as described by Ruberto et al. (2001) with some modifications [15]. The ABTS radical cation was produced by reaction of a 7 mM stock solution of ABTS with 2.45 mM potassium persulfate and the mixture was allowed to stand in the dark at room temperature for 12 h before use. The ABTS solution was diluted with methanol to give an absorbance of 0.7 ± 0.01 at 734 nm. Grape marmalade extracts were allowed to react with 2 mL of the ABTS solution, and the absorbance was measured at 734 nm after 1 min. Trolox was used as a reference compound. The results were expressed as Trolox equivalent antioxidant capacity values and calculated as mean value ± standard deviation (SD) (*n* = 3).

#### 2.5.4. Tests of DPPH

The procedures of Tagliazucchi were adopted to determine DPPH scavenging activity; therefore, 1 mL of grape marmalade supernatant and 5 mL of newly prepared 0.1 mM DPPH methanol solution were combined and maintained in the dark for 1 h [18]. A spectrophotometer (MODEL SP-UV1100, Sigma-Aldririch, CA, USA) was used to measure the absorbance at 517 nm. Thus, the grape marmalade supernatant with methanol (1 mL) was replaced by a blank to calculate the proportion of DPPH as follows:

DPPH (%) = (1 − (A517 sample/A517 blank)) × 100%(2)

where A517 is the absorbance at 517 nm.

### 2.6. Enumeration of Probiotic Bacteria

Aliquots of both control and GM samples, inoculated and non-inoculated from the original isolates, were evaluated at regular intervals of 0, 15, 30, 45, and 90 days at 4 and 25 °C of storage, for enumeration of viable bacteria according to the procedure of Randazzo [14].

### 2.7. Statistical Analysis

All data were submitted to ANOVA tests for statistical analysis. Then, the data were compared via the statistical procedure of SPSS and the Tukey test (*p* < 0.05).

## 3. Results

### 3.1. Physicochemical Parameters of Grape Control Media and Marmalade Samples

Lactic acid is the main product of many food fermentations; it is formed by microbial degradation of sugars in products. It decreases the pH to unfavorable levels in fermentation for growth of spoilage organisms [19]. Lactic acids were found as end-products in probiotic grape marmalade (Table 1). Lactic acid was found at the highest concentration compared to acetic acid. It showed results of 44.6 ± 11 mM (*L. plantarum* MF369875.1), 39.8 ± 9 mM (*W. paramesenteroides* CP023501.1), 41.4 ± 8 mM (*E. faecalis* HQ802261.1), and 42.7 ± 8 mM (*L. paraplantarum* AB362736.1).

On the other hand, acetic acid, which is a byproduct of the lactic-acid fermentation, has an organoleptic impact on the matrix, and its preservative action even at identical pH levels is greater than that of lactic acid [19]. The acetic acids showed concentrations of 1.6 ± 0.4 mM (*L. plantarum* MF369875.1), 4.7 ± 0.7 mM (*W. paramesenteroides* AB362736.1), 3.2 ± 1.3 mM (*E. faecalis* HQ802261.1), and 2.2 ± 0.8 mM (*L. paraplantarum* AB362736.1).

In Table 2, the pH values in GCM of inoculated and non-inoculated samples are shown with the four isolates at both room and refrigerated storages. Overall, reduction in pH of all samples persisted at 4 °C of storage. Interestingly, the inoculated GCM samples with wild-type strains exhibited identical pH values at 25 °C to other reports. The synthetic medium inoculated with selected isolates indicated a particularly substantial reduction at 25 °C of storage attaining pH 3 to 3.4 within 60 days. Thus, a distinct trend of pH was noticed in the synthetic medium with wild-type isolates in refrigerated conditions. The grape synthetic medium samplings inoculated with isolates *L. plantarum* MF369875.1*, W. paramesenteroides* CP023501.1, and *E. faecalis* HQ802261.1 exhibited significant pH values during storage; however, the synthetic medium inoculated with the strain *L. paraplantarum* AB362736.1 had fairly regular pH levels.

**Figure 1:**
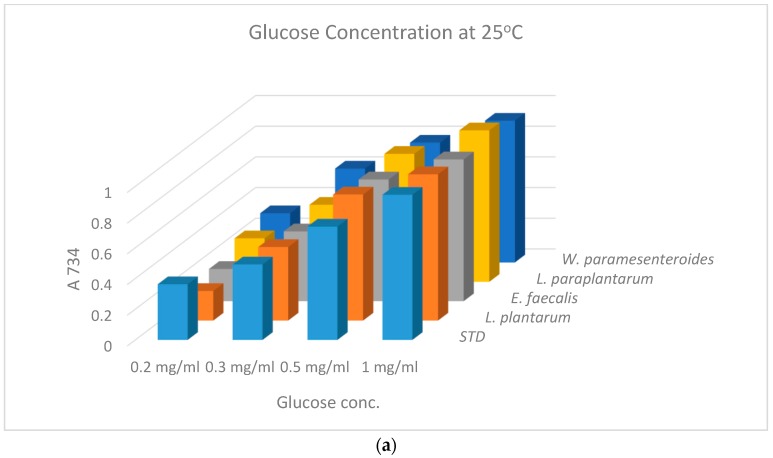

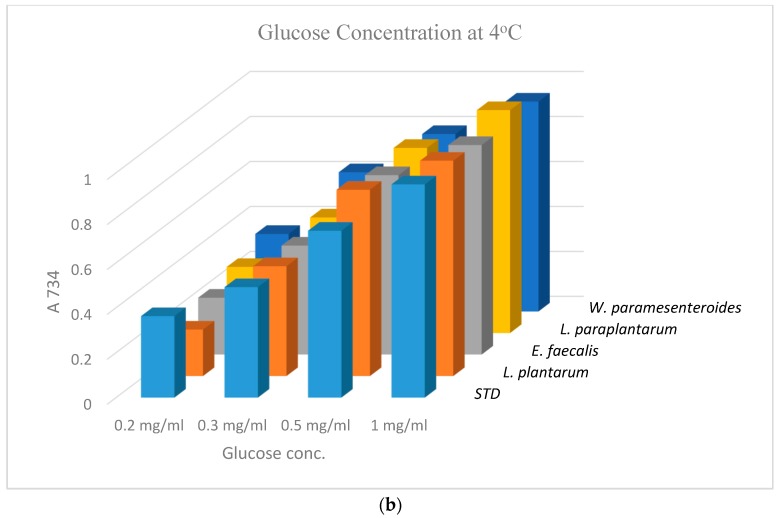
Glucose concentration of the marmalade at different temperatures of storage: 4 °C and 25 °C.

The sugar concentration in grape marmalade was evaluated to 100 mg/g of glucose until the end of preservation. The results of non-inoculated and inoculated grape marmalades are shown in Figure 1, and sugar composition was 0.339 g of glucose at A734 for the freshly prepared samples. Jams and marmalades basically constitute fruit peel, sugar, and water; in addition, the more bitter the peel is, the better the flavor of the marmalade will be. It represents a beneficial origin of dietary fibers, vitamins, and minerals. These nutrients possess healthy benefits in several ways considering when they are incorporated with probiotic bacteria and antioxidants.

### 3.2. Viability of Probiotic Bacteria in GCM and GM at Storage

The viability of probiotic strains in storage was investigated in the synthetic medium and marmalade of grapes at refrigerated and room temperatures (Table 3). Samples of non-inoculated grape synthetic medium and marmalade exhibited slight numbers in the media during preservation at room and refrigerated conditions. Generally, synthetic medium samples inoculated with the wild-type *W. paramesenteroides* CP023501.1*, E. faecalis* HQ802261.1, and *L. plantarum* MF369875.1 indicated strong growth within 30 days of storage at room temperature, whereas specimens inoculated with *L. paraplantarum* AB362736.1 showed seemingly constant values (Table 3). All synthetic medium samples indicated a substantial reduction in survival over the crucial range of 10^6^ CFU/g for more than 45 days. Nevertheless, a distinct tendency was noticed at 4 °C; most initial isolates showed a reduction after 30 days (log 8 CFU/mL) of preservation and growth within 45 days. Strains of *W. paramesenteroides* CP023501.1*, E. faecalis* HQ802261.1, and *L. plantarum* MF369875.1 remained viable in the grape synthetic medium during 90 days of storage, while subsisting beyond the crucial counts of 10^6^ CFU/g. Most of the initial isolates exhibited strong survival with considerable growth in 30 days at room temperature, according to cell density ranging from 10^10^ to 10^12^ CFU/g. Most isolates excluding *E. faecalis* HQ802261.1 had counts over the crucial standard of 10^6^ CFU/g for more than 60 days at 25 °C (Table 3). Thus, most of the isolates stayed active above 10^7^ CFU/g in the grape marmalade samplings kept in refrigerated conditions. Under those terms, all initial isolates indicated significant counts (proximate to 10^10^ CFU/g) from 15 to 45 days of storage, excluding *W. paramesenteroides* CP023501.1 and *E. faecalis* HQ802261.1, which maintained the greatest values after 60 days of storage.

### 3.3. Correlation and Properties of the Probiotic Product

The isolates of *W. paramesenteroides* CP023501.1*, E. faecalis* HQ802261.1*, L. paraplantarum* AB362736.1, and *L. plantarum* MF369875.1 performed well in sterile grape marmalade with no addition of nutrients. Table 2 and Table 3 summarize the evolution of lactic inoculation time of grape marmalade by *W. paramesenteroides* CP023501.1*, E. faecalis* HQ802261.1, *L. paraplantarum* AB362736.1, and *L. plantarum* MF369875.1. The counts of *W. paramesenteroides* CP023501.1*, E. faecalis* HQ802261.1, and *L. plantarum* MF369875.1 attained 10^9^ CFU/mL in grape marmalade after three days of storage at 30 °C (Table 3). The extension of the development time beyond 15 days showed no significant decrease in the cell counts of all the tested bacteria. Both *L. plantarum* MF369875.1 and *W. paramesenteroides* CP023501.1 generated more lactic acid than *L. paraplantarum* AB362736.1. In reference, *L. plantarum* MF369875.1 and *W. paramesenteroides* CP023501.1 generated about 1% lactic acid after three days at 30 °C. In identical development terms, *L. paraplantarum* AB362736.1 generated a titratable acidity of 0.79% lactic acid. Thus, only *L. paraplantarum* AB362736.1 failed to significantly correlate with other strains at 4 °C for the counts in the grape marmalade (Table 4); however, most isolates showed a certain positive correlation at some points with a *p*-value = 0.01 or *p*-value = 0.05. Nonetheless, a different case was observed for the acidity of the strains in room and refrigerated conditions. At *p*-values 0.01 and 0.05, none of the isolates showed significant differences (Table 5), since *E. faecalis* HQ802261.1 and *L. plantarum* MF369875.1 exhibited significant correlation of the strains at 25 °C and 4 °C.

### 3.4. Antioxidant Characteristics of Probiotic Grape Marmalade

The antioxidant influence of LAB, in terms of the antioxidant capability of cells and the intracellular cell-free extract of LAB, was investigated based on several antioxidative tests. In this research, the antioxidative activity of grape marmalade is shown with various cultures of *W. paramesenteroides* CP023501.1*, E. faecalis* HQ802261.1*, L. paraplantarum* AB362736.1, and *L. plantarum* MF369875.1 (Table 6). In particular, according to the cultures applied, the fermented grape marmalade exhibited a strong antioxidant capability, estimated as total antioxidative activity, ABTS, and DPPH. Nonetheless, the probiotic grape marmalade that contained *W. paramesenteroides* CP023501.1 indicated an antioxidative activity of 77% that varied insignificantly from that of probiotic grape marmalade with *L. paraplantarum* AB362736.1 and *L. plantarum* MF369875.1 (72% and 71%, respectively) within three days of fermentation.

Antioxidative activity decreased with *E. faecalis* HQ802261.1 after fermentation (15.3% versus 8.7%), regarding a decrease of 7% over 90 days. An apparent decrease in antioxidative activity was observed in the grape marmalade using *L. paraplantarum* AB362736.1 (decrease by about 5%). The ABTS in the probiotic grape marmalade with LAB exhibited no significant differences during storage in room and refrigerated conditions. GM fermented with *E. faecalis* and *W. paramesenteroides* CP023501.1 exhibited higher ABTS (69%) than *L. plantarum* MF369875.1 and *L. paraplantarum* AB362736.1, whose inhibiting powers were 67.2 and 64.7, respectively. Similar findings were obtained for DPPH, where the probiotic grape marmalade with *W. paramesenteroides* CP023501.1 exhibited the highest scavenging activity (73%). The DPPH capacity of grape marmalade decreased, notably with the probiotic *L. paraplantarum* AB362736.1, after 90 days of storage at room temperature.

## 4. Discussion

The cholesterol content and lactose non-tolerance constitute two substantial challenges found with milk-based products. In addition, there is an increase of vegetarianism in some developed countries, as well as of demand for non-dairy probiotic products [20]. Numerous studies recently showed that certain raw materials are capable of introducing new non-dairy probiotic products into the market [21,22]. Thus, proper studies of non-dairy probiotic products focused on the numerous fundamental and external properties of foods, such as pH, nutrient accessibility, glucose content, O2 level, water, and temperature, showing how they affect the survival of probiotic cells [23,24,25]. In this research, the survival of four different wild strains was studied in grape marmalade in room and refrigerated conditions of storage. All the isolates were investigated for their characteristics, viability at pH 2, strong tolerance to bile salts, resistance to different antibiotics, such as vancomycin, chloramphenicol, tetracycline, ampicillin, and penicillin, and their antibacterial trend against *Staphylococcus aureus, Listeria monocytogenes*, and *Escherichia coli*. This study was able to show that the alleged wild probiotic strains survived in the grape marmalade during storage, thus indicating that this vehicle might be a potential candidate as a probiotic medium. Thus, the isolates exhibited significantly higher viability in grape marmalade than in the synthetic grape medium, which confirmed that food preparations can affect probiotic survival in storage. Some authors argued that a strong matrix can preserve the strains during storage; thus, in this research, the grape marmalade preparation with its innate constituents apparently promoted the survival of the probiotics [26,27,28]. Previous researches demonstrated the viability of probiotic strains in highly acidic food matrices during storage (4–5 °C), claiming that the increase and survival of cells in some fruits and vegetables may be subject to the strains applied [29,30]. In this research, the survival of the strains confirmed the results shown by previous studies, which showed the probiotic strain cells had comparative stability in milk compared to non-dairy products.

The changes in the stability of isolates were attributed to pH and temperature during storage. Champagne reported that, in several fermented milk byproducts, the deficit of probiotic strain survival was assigned to a reduction of pH (pH 4–5) and to the production of organic acids as a result of fermentation [14]. Sheehan indicated that the isolates demonstrated strong survival at pH 4, and indicated that the control strain might survive for 12 weeks at 5 °C in orange juice (pH 3.65) and in pineapple juice at pH 3.4 [31]. This research reported that most of the isolates stayed active beyond the crucial count in grape marmalade at room temperature within 45 days. The correlation of bacteria numbers with pH levels confirmed the survival of isolates, related to previous statistics, while the survival of the wild strains was to a lesser degree. In addition, the results indicated an insignificant increase in glucose concentrations in 25 °C storage. The increase in glucose might be due to two factors that occurred concurrently: intracellular bacterial growth and sucrose inversion, which was precipitated at low pH levels and at high storage temperatures. Essentially, marmalades and jams are considered as highly stable products, due to their low pH, glucose concentration, and their A_w_; they constitute suitable media for sucrose inversion [32,33]. Acetic acid has a pungent and penetrating odor detectable at low concentrations (0.48 to 1 ppm) in products. The United States (US) organoleptic threshold level for acetic acid is 10 ppm. Indeed, acetic acid is the main constituent of vinegar after water, since it provides its acidic taste and its pungent odor detectable from 1 ppm. Its concentration in contaminated wines is 0.75 g/L and its organoleptic threshold level is 0.4 to 0.75 g/L. A wine is said to be “marketable” if it does not exceed 0.9 g/L. In red wine, it appears in low concentrations; the organoleptic threshold level of this molecule is 0.16 g/L, but it can even initiate the denaturation of the aromas of a wine below this value (loss of fruitiness) [34,35].

This study also highlighted the antioxidative effect of lactic acid bacteria as of great interest for researchers [36,37]. The antioxidative effect of grape marmalade is presented in Table 3, using different strains of *W. paramesenteroides* CP023501.1*, E. faecalis* HQ802261.1*, L. paraplantarum* AB362736.1, and *L. plantarum* MF369875.1. Based on the strains applied, the grape marmalade demonstrated strong antioxidant capacity, evaluated as DPPH scavenging activity and total antioxidant activity.

Similar findings were exhibited for DPPH; grape marmalade fermented with *L. plantarum* MF369875.1 and *W. paramesenteroides* CP023501.1 showed the highest scavenging activity at 70%, whereas *L. paraplantarum AB362736.1* reduced the DPPH of grape marmalade within 30 days at room temperature. In a study conducted by Chen et al. (2008), it was observed that the fermentation of carbohydrates by innate intestinal LAB exhibited strong antioxidant effects. Table 2 indicates that the glucose concentration of the grape marmalade was probably a sufficient and essential carbon source for *Lactobacillus* strains [38].

The metabolism of the bacteria apparently affected the capacity of the antioxidant activity in the marmalade according to the strains used. Some researchers reported the effect of fermentation on the food’s antioxidative properties. For instance, Espinoza et al. (2010) reported the antioxidative characteristics of fermented probiotic carrot juice [22]; it was reported that sugar apple as a substrate indicated no significant difference between fresh and fermented juices for the strains of *L. delbrueckii, L. paracasei*, *and L. casei.* Fermented sugar apple juice showed 65–75% antioxidant activity and DPPH free-radical scavenging of 72% [39]. The fermented grape marmalade showed antioxidative activities, which changed with the strains applied, but *L. plantarum* MF369875.1 and *L. paraplantarum* AB362736.1 had no effect on the antioxidative activity of fermented grape marmalade compared to non-fermented synthetic medium. In this study, the fermentation of grape marmalade with *W. paramesenteroides* CP023501.1 showed better antioxidative activity than the non-fermented synthetic medium (Table 6). As a result, the antioxidative activity changed with the starters used. Furthermore, the accumulation of intracellular sucrose in response to osmotic stress may explain the reduction of sucrose; however, this assumption needs to be studied in more detail [14]. The findings of this study showed that *L. plantarum* MF369875.1 and *W. paramesenteroides* CP023501.1 were the best probiotic bacteria to produce non-dairy probiotic products. Moreover, the antioxidant ability exhibited for 72 h of the fermentation period by *E. faecalis* showed no significant difference comparing to *L. paraplantarum* AB362736.1*, L. plantarum* MF369875.1, and *W. paramesenteroides* CP023501.1 [11]. Grape marmalade might be a good candidate for the production of new functional probiotics, which might efficiently subsist probiotic strains of *Lactobacillus* both under refrigerated conditions and at room temperature. A quotidian absorption of approximately 10 g of probiotic marmalade might provide from 10^8^ to 10^9^ viable probiotic bacteria, with stored preparations for 40–90 days at both 25 °C and 4 °C [4]. Similarly, the results of this study correlate with those in fermented dairy products, with over 10^6^ CFU/mL within 30 days of storage at 4 °C.

## 5. Conclusions

In summary, this study aimed to develop a probiotic marmalade and determine the antioxidant activity of this product at storage. Due to the growth and viability of the probiotics in the product during storage, the antioxidative capacity of the marmalade increased considerably. It is reported that probiotic bacteria have the ability to break down long chains of protein and lipid, thus increasing the capacity of antioxidants. The development of antioxidant activity was strain-specific. It is highlighted that lipid peroxidation inhibition is related to the bacterial growth. As a result, the development of probiotic bacteria prevents the accumulation of intracellular sucrose in response to osmotic stress caused by the action of bacteria in the marmalade. The formation of the inhibitory activity of sucrose oxidation by *L. paraplantarum* AB362736.1*, L. plantarum* MF369875.1*, W. paramesenteroides* CP023501.1, and *E. faecalis* HQ802261.1 was more related to the bacterial growth in marmalade. Therefore, the development and viability of probiotic bacteria in marmalade led to this increase in the antioxidant capacity of the non-dairy product. Thus, this non-dairy probiotic grape marmalade may be a source of polyphenolic compounds providing an alternative option for people who are intolerant to lactose in dairy products.

## Figures and Tables

**Table 1:** Concentrations (mM) of organic lactic and acetic acids after growth (48 h at 37 °C) of selected *Lactobacillus* strains.

Strains	Lactic Acid (mM)	Acetic Acid (mM)
*Lactobacillus plantarum* MF369875.1	44.6 ± 11 ^a^	1.6 ± 0.4 ^b^
*Weissella paramesenteroides* CP023501.1	39.8 ± 9 ^b^	4.7 ± 0.7 ^a^
*Eenterococcus faecalis* HQ802261.1	41.4 ± 8 ^b^	3.2 ± 1.3 ^a^
*L. paraplantarum* AB362736.1	42.7 ± 8 ^b^	2.2 ± 0.8 ^b^

Each value is expressed as the mean ± standard deviation (*n*_¼_ = 3) analyzed in duplicate. ^a,b^ Values in the same column with different superscript letters differ significantly (*p* < 0.05).

**Table 2:** pH values about bacteria counts for the non-inoculated and inoculated grape control media (GCM) and grape marmalade (GM) kept at room and refrigerated temperatures.

**pH Values in Grape Control Media (GCM)**						
**Temp. (°C)**	**Time (days)**	**Control**	***L. plantarum***	***E. faecalis***	***L. paraplantarum***	***W. paramesenteroides***
25 °C	0	4.10 ± 0.03 ^a^	4.12 ± 0.08 ^a^	4.03 ± 0.08 ^a^	3.97 ± 0.12 ^a^	4.13 ± 0.13 ^a^
	15	3.82 ± 0.04 ^a^	3.77 ± 0.10 ^ab^	3.70 ± 0.07 ^ab^	3.73 ± 0.07 ^a^	3.69 ± 0.09 ^ab^
	30	3.79 ± 0.06 ^a^	3.75 ± 0.04 ^a^	3.72 ± 0.04 ^a^	3.71 ± 0.09 ^a^	3.69 ± 0.05 ^ab^
	60	3.70 ± 0.08 ^ab^	3.64 ± 0.06 ^b^	3.67 ± 0.13 ^ab^	3.64 ± 0.09 ^b^	3.72 ± 0.02 ^a^
	90	3.52 ± 0.09 ^ab^	3.47 ± 0.05 ^b^	3.52 ± 0.11 ^b^	3.49 ± 0.04 ^b^	3.45 ± 0.08 ^b^
4 °C	0	4.08 ± 0.10 ^a^	4.05 ± 0.08 ^a^	4.02 ± 0.11 ^a^	3.98 ± 0.09 ^ab^	4.07 ± 0.13 ^a^
	15	3.81 ± 0.07 ^a^	3.79 ± 0.04 ^a^	3.75 ± 0.14 ^a^	3.81 ± 0.04 ^a^	3.85 ± 0.14 ^a^
	30	3.71 ± 0.06 ^ab^	3.68 ± 0.16 ^ab^	3.70 ± 0.06 ^ab^	3.73 ± 0.06 ^a^	3.74 ± 0.06 ^a^
	60	3.73 ± 0.08 ^ab^	3.69 ± 0.08 ^ab^	3.66 ± 0.08 ^ab^	3.70 ± 0.09 ^a^	3.70 ± 0.08 ^a^
	90	3.54 ± 0.09 ^b^	3.53 ± 0.07 ^ab^	3.54 ± 0.09 ^b^	3.50 ± 0.07 ^ab^	3.52 ± 0.09 ^b^
**pH Values in Grape Marmalade (GM)**						
**Temp. (°C)**	**Time (days)**	**Control**	***L. plantarum***	***E. faecalis***	***L. paraplantarum***	***W. paramesenteroides***
25 °C	0	4.08 ± 0.12 ^a^	4.05 ± 0.10 ^a^	3.91 ± 0.14 ^a^	3.85 ± 0.10 ^a^	3.78 ± 0.17 ^a^
	15	3.83 ± 0.07 ^ab^	3.76 ± 0.04 ^a^	3.77 ± 0.24 ^ab^	3.71 ± 0.17 ^a^	3.68 ± 0.16 ^ab^
	30	3.79 ± 0.16 ^a^	3.75 ± 0.16 ^a^	3.72 ± 0.16 ^ab^	3.70 ± 0.06 ^a^	3.69 ± 0.16 ^ab^
	60	3.75 ± 0.08 ^ab^	3.68 ± 0.18 ^ab^	3.67 ± 0.08 ^ab^	3.70 ± 0.14 ^a^	3.67 ± 0.18 ^ab^
	90	3.53 ± 0.12 ^ab^	3.49 ± 0.09 ^b^	3.51 ± 0.12 ^b^	3.49 ± 0.09 ^ab^	3.58 ± 0.09 ^b^
4 °C	0	4.07 ± 0.09 ^a^	4.02 ± 0.18 ^a^	3.97 ± 0.13 ^a^	3.95 ± 0.08 ^a^	3.90 ± 0.11 ^a^
	15	3.85 ± 0.34 ^a^	3.80 ± 0.24 ^a^	3.77 ± 0.31 ^a^	3.81 ± 0.17 ^a^	3.81 ± 0.23 ^a^
	30	3.71 ± 0.25 ^a^	3.71 ± 0.16 ^ab^	3.69 ± 0.21 ^ab^	3.71 ± 0.18 ^a^	3.70 ± 0.21 ^a^
	60	3.68 ± 0.17 ^ab^	3.65 ± 0.17 ^a^	3.63 ± 0.14 ^ab^	3.69 ± 0.41 ^a^	3.69 ± 0.18 ^a^
	90	3.55 ± 0.29 ^b^	3.52 ± 0.15 ^b^	3.52 ± 0.18 ^ab^	3.51 ± 0.12 ^ab^	3.52 ± 0.13 ^ab^

Formulation of values as the average ± SD in triplicate runs. Different letters in the same column indicate significant differences with a *p*-value greater than 0.05.

**Table 3:** LAB counts (log CFU/g) for the non-inoculated and inoculated GCM and GM kept at room and refrigerated temperatures.

**Mean Counts (log CFU/g) Grape Control Media (GCM)**						
Temp. (°C)	Time (days)	Control	*L. plantarum*	*E. faecalis*	*L. paraplantarum*	*W. paramesenteroides*
25 °C	0	8.90 ± 0.13 ^a^	8.73 ± 0.27 ^a^	8.81 ± 0.18 ^a^	8.75 ± 0.23 ^a^	8.84 ± 0.21 ^a^
	15	9.22 ± 0.74 ^a^	9.01 ± 0.69 ^a^	9.19 ± 0.71 ^a^	9.26 ± 0.72 ^a^	9.89 ± 0.68 ^a^
	30	8.75 ± 0.86 ^a^	7.98 ± 0.23 ^a^	6.75 ± 0.37 ^a^	8.67 ± 0.89 ^a^	8.42 ± 0.85 ^a^
	60	8.72 ± 0.48 ^a^	6.25 ± 0.51 ^b^	4.98 ± 0.63 ^c^	6.28 ± 0.49 ^b^	7.21 ± 0.52 ^a^
	90	7.44 ± 0.29 ^a^	5.71 ± 0.23 ^c^	3.74 ± 0.21 ^c^	5.74 ± 0.54 ^b^	5.77 ± 0.18 ^b^
4 °C	0	8.92 ± 0.24 ^a^	8.77 ± 0.26 ^a^	8.85 ± 0.21 ^a^	8.98 ± 0.28 ^a^	8.72 ± 0.17 ^a^
	15	9.26 ± 0.73 ^a^	9.17 ± 0.71 ^a^	9.09 ± 0.70 ^a^	9.19 ± 0.68 ^a^	8.53 ± 0.74 ^a^
	30	8.80 ± 0.93 ^a^	8.38 ± 0.86 ^a^	8.61 ± 0.91 ^a^	8.68 ± 0.87 ^a^	8.75 ± 0.91 ^a^
	60	8.68 ± 0.62 ^a^	7.41 ± 0.49 ^a^	9.02 ± 0.54 ^a^	8.08 ± 0.51 ^a^	9.01 ± 0.59 ^a^
	90	7.48 ± 0.57 ^a^	6.17 ± 0.35 ^b^	7.51 ± 0.56 ^a^	7.50 ± 0.60 ^a^	6.77 ± 0.11 ^a^
**Mean Counts (log CFU/g) Grape Marmalade (GM)**						
Temp. (°C)	Time (days)	Control	*L. plantarum*	*E. faecalis*	*L. paraplantarum*	*W. paramesenteroides*
25 °C	0	8.92 ± 014 ^a^	8.70 ± 0.24 ^a^	8.79 ± 0.21 ^a^	9.02 ± 0.69 ^a^	8.81 ± 0.45 ^a^
	15	9.13 ± 0.82 ^a^	9.07 ± 0.75 ^a^	9.04 ± 0.77 ^a^	8.86 ± 0.67 ^a^	9.06 ± 0.71 ^a^
	30	8.78 ± 0.50 ^a^	8.75 ± 0.59 ^a^	7.80 ± 0.53 ^a^	8.71 ± 0.82 ^a^	8.71 ± 0.46 ^a^
	60	9.08 ± 0.61 ^a^	8.98 ± 0.56 ^a^	8.88 ± 0.59 ^a^	8.75 ± 0.67 ^a^	8.95 ± 0.89 ^a^
	90	6.84 ± 0.53 ^a^	7.17 ± 0.48 ^a^	6.84 ± 0.52 ^a^	6.77 ± 0.12 ^a^	7.73 ± 0.36 ^a^
4 °C	0	8.93 ± 0.38 ^a^	8.77 ± 0.66 ^a^	8.78 ± 0.37 ^a^	9.10 ± 0.84 ^a^	8.86 ± 0.3 ^a^
	15	9.16 ± 0.85 ^a^	9.29 ± 0.92 ^a^	9.19 ± 0.89 ^a^	9.26 ± 0.98 ^a^	9.14 ± 0.81 ^a^
	30	8.78 ± 0.34 ^a^	8.83 ± 0.78 ^a^	8.75 ± 0.41 ^a^	8.85 ± 0.79 ^a^	8.85 ± 0.33 ^a^
	60	8.75 ± 0.28 ^a^	9.11 ± 0.96 ^a^	8.95 ± 0.19 ^a^	9.15 ± 0.96 ^a^	9.11 ± 0.93 ^a^
	90	7.46 ± 0.19 ^a^	8.74 ± 0.58 ^a^	7.84 ± 0.30 ^a^	8.86 ± 0.87 ^a^	7.81 ± 0.13 ^a^

Formulation of values as the average ± SD in triplicate runs. Different letters in the same column indicate significant differences with a *p*-value greater than 0.05.

**Table 4:** Pearson’s correlation coefficients of *Lactobacillus* counts in the grape marmalade at both 25 °C and 4 °C.

T	Organisms	Control	*L. plantarum*	*E. faecalis*	*L. paraplantarum*	*W. paramesenteroides*	*Control*	*L. plantarum*	*E. faecalis*	*L. paraplantarum*	*W. paramesenteroides*
25 °C	Control	1									
	*L. plantarum*	0.995 **	1								
	*E. faecalis*	0.913 *	0.900 *	1							
	*L. paraplantarum*	0.984 **	0.965 **	0.882 *	1						
	*W. paramesenteroides*	0.993 **	0.995 **	0.938 *	0.964 **	1					
4 °C	*Control*	0.978 **	0.972 **	0.898 *	0.978 **	0.977 **	1				
	*L. plantarum*	0.588	0.646	0.691	0.453	0.672	0.581	1			
	*E. faecalis*	0.974 **	0.985 **	0.927 *	0.932 *	0.992 **	0.972 **	0.743	1		
	*L. paraplantarum*	0.672	0.673	0.902 *	0.606	0.740	0.681	0.811	0.765	1	
	*W. paramesenteroides*	0.992 **	0.998 **	0.919 *	0.954 *	0.997 **	0.963 **	0.678	0.988 **	0.711	1

** Values are significantly correlated at *p*-value = 0.01; * values are significantly correlated at *p*-value = 0.05; T: temperature.

**Table 5:** Pearson’s correlation coefficients of the pH values in the grape marmalade at both 25 °C and 4 °C.

T	Organisms	Control	*L. plantarum*	*E. faecalis*	*L. paraplantarum*	*W. paramesenteroides*	Control	*L. plantarum*	*E. faecalis*	*L. paraplantarum*	*W. paramesenteroides*
**25 °C**	Control	1									
	*L. plantarum*	0.996 **	1								
	*E. faecalis*	0.991 **	0.990 **	1							
	*L. paraplantarum*	0.976 **	0.959 **	0.940 *	1						
	*W. paramesenteroides*	0.991 **	0.990 **	0.966 **	0.984 **	1					
**4 °C**	Control	0.985 **	0.991 **	0.997 **	0.925 *	0.962 **	1				
	*L. plantarum*	0.983 **	0.984 **	0.999 **	0.924 *	0.954 *	0.997 **	1			
	*E. faecalis*	0.984 **	0.988 **	0.999 **	0.925 *	0.958 *	0.999 **	0.999 **	1		
	*L. paraplantarum*	0.985 **	0.971 **	0.987 **	0.961 **	0.959 **	0.973 **	0.983 **	0.979 **	1	
	*W. paramesenteroides*	0.968 **	0.947 *	0.970 **	0.953 *	0.938 *	0.950 *	0.966 **	0.959 **	0.996 **	1

** Values are significantly correlated at *p*-value = 0.01; * values are significantly correlated at *p*-value = 0.05; T: temperature.

**Table 6:** Antioxidative properties of the grape marmalade (percentage inhibition). NO—nitric oxide; ABTS—2,2’-azino-bis(3-ethylbenzothiazoline-6-sulfonic acid); DPPH—2,2-diphenyl-1-picrylhydrazyl.

NO Radical (% at Absorbance = 534 nm)					ABTS (% at Absorbance = 700 nm)				DPPH (% at Absorbance = 340 nm)			
Time (Day)	*L. plantarum*	*E. Faecalis*	*L. para plantarum*	*W. paramesenteroides*	*L. plantarum*	*E. faecalis*	*L. paraplantarum*	*W. paramesenteroides*	*L. plantarum*	*E. Faecalis*	*L. paraplantarum*	*W. paramesenteroides*
**0** **15** **30** **45** **60** **90** **GSM**	15.3 ± 0.8 ^a^ 16.6 ± 0.5 ^a^ 14.8 ± 0.7 ^a^ 13.7 ± 0.4 ^a^ 12.9 ± 0.4 ^a^ 8.7 ± 0.3 ^b^ 14.8 ± 0.5 ^a^	14.4 ± 0.9 ^a^ 13.7 ± 0.7 ^a^ 13.6 ± 0.7 ^a^ 11.8 ± 0.6 ^a^ 12.9 ± 0.7 ^a^ 8.7 ± 0.5 ^b^	13.1 ± 0.9 ^a^ 14.5 ± 0.8 ^a^ 12.1 ± 0.8 ^a^ 11.9 ± 0.7 ^a^ 10.4 ± 0.8 ^b^ 8.6 ± 0.6 ^b^	14.9 ± 1.1 ^a^ 16.3 ± 0.9 ^a^ 14.4 ± 0.8 ^a^ 12.8 ± 0.7 ^a^ 11.7 ± 0.8 ^a^ 9.8 ± 0.6 ^b^	67.2 ± 2.4 ^a^ 65.5 ± 1.9 ^a^ 63.3 ± 1.6 ^a^ 62.4 ± 1.7 ^a^ 59.7 ± 1.1 ^b^ 54.2 ± 1.2 ^c^ 66.3 ± 2.2 ^a^	69.2 ± 1.6 ^a^ 67.5 ± 1.5 ^a^ 64.6 ± 1.5 ^a^ 62.4 ± 1.6 ^a^ 60.9 ± 1.4 ^b^ 58.7 ± 1.3 ^b^	64.7 ± 1.3 ^a^ 62.5 ± 1.4 ^a^ 59.6 ± 1.3 ^b^ 57.2 ± 1.4 ^b^ 55.4 ± 1.2 ^c^ 53.5 ± 1.1 ^c^	69.8 ± 1.2 ^a^ 68.5 ± 1.1 ^a^ 67.2 ± 1.2 ^a^ 64.3 ± 1.1 ^a^ 62.7 ± 1.3 ^a^ 59.4 ± 1.2 ^b^	72.9 ± 3.1 ^a^ 72.1 ± 2.9 ^a^ 70.4 ± 3.2 ^a^ 68.8 ± 1.2 ^a^ 66.9 ± 1.8 ^a^ 64.7 ± 1.9 ^b^ 71.8 ± 2.2 ^a^	73.1 ± 3.4 a 72.8 ± 3.2 a 71.9 ± 2.3 a 69.9 ± 1.8 a 67.4 ± 1.6 a 65.3 ± 1.8 b	71.4 ± 2.5 ^a^ 70.7 ± 1.9 ^a^ 68.7 ± 1.3 ^a^ 67.3 ± 1.1 ^a^ 65.8 ± 1.5 ^b^ 63.6 ± 1.3 ^b^	73.4 ± 3.1 ^a^ 72.7 ± 3.0 ^a^ 70.6 ± 2.3 ^a^ 68.8 ± 1.8 ^a^ 67.9 ± 2.1 ^a^ 66.7 ± 2.1 ^a^

Formulation of values as the average ± SD in triplicate runs. Values with similar letters showed non-significant differences with a *p*-value greater than 0.05.
